# Food-web complexity, meta-community complexity and community stability

**DOI:** 10.1038/srep24478

**Published:** 2016-04-13

**Authors:** A. Mougi, M. Kondoh

**Affiliations:** 1 Department of Biological Science, Faculty of Life and Environmental Science, Shimane University, 1060 Nishikawatsu-cho, Matsue 690-8504, Japan; 2 Department of Environmental Solution Technology, Faculty of Science and Technology, Ryukoku University, 1-5 Yokoya, Seta Oe-cho, Otsu 520-2194, Japan

## Abstract

What allows interacting, diverse species to coexist in nature has been a central question in ecology, ever since the theoretical prediction that a complex community should be inherently unstable. Although the role of spatiality in species coexistence has been recognized, its application to more complex systems has been less explored. Here, using a meta-community model of food web, we show that meta-community complexity, measured by the number of local food webs and their connectedness, elicits a self-regulating, negative-feedback mechanism and thus stabilizes food-web dynamics. Moreover, the presence of meta-community complexity can give rise to a positive food-web complexity-stability effect. Spatiality may play a more important role in stabilizing dynamics of complex, real food webs than expected from ecological theory based on the models of simpler food webs.

In the 1950s, Charles Elton stated that simplified ecosystems, such as agricultural or degraded ecosystems, are more prone to population oscillations than natural complex ecosystems. He further hypothesized that community complexity may beget ecosystem stability^1^. However, this inference was challenged by theoretical studies of the stability of ‘random’ community models, which concluded that more complex communities are less likely to be stable^2^,^3^,^4^. Thus, an apparent gap between theory and observation emerged. Ecological factors that explain the persistence of ‘theoretically unstable’ complex communities in nature remain a central topic in ecology. Hypothesized factors include a ‘nonrandom’ community network structure, such as of interaction strength or sign distributions within a network, and flexibility in trophic links^5^,^6^,^7^,^8^,^9^,^10^,^11^,^12^,^13^. Numerous nonrandom and stabilizing structural properties have been identified via analyses of real food webs. Nevertheless, it remains unclear how widely those features are realized in diverse natural ecosystems^14^,^15^,^16^.

Here, we propose that spatial heterogeneity alone, as a general and inherent feature of any natural ecosystem, can elicit a positive complexity-stability effect. Our world is undoubtedly spatial, and species interactions are spatially limited^17^. Stabilizing effects of spatiality on prey-predator population dynamics have been well recognized and studied both theoretically and empirically^18^,^19^,^20^. However, those studies considered simple communities consisting of only a few species^17^,^21^,^22^, did not account for spatial effects^23^,^24^, did not examine interactions between food-web complexity and spatiality^25^ or food-web complexity is only realized as a result of spatial aggregation of local simple sub-webs^26^,^27^,^28^. Thus, how essential spatiality may be for the persistence of more complex food webs is still not clear. We aim to reveal that the incorporation of spatiality may totally change, and even reverse, our understanding of how ecosystem complexity affects ecosystem stability.

Consider a meta-community in which organisms randomly move between numerous coexisting local food webs. This ‘meta-food web’ can be viewed as a network of networks and, thus, characterized by two different kinds of network complexity: food-web complexity and meta-community complexity. Food-web complexity can be quantified by the number of species involved in a local food web, *N*, and the probability that a pair of species is connected by a trophic link, *P*. Similarly, meta-community complexity can be quantified by the number of local food webs, *H*_*N*_, and the proportion of food-web pairs between which an organism can move, *H*_*P*_ (a proportion of realized link to all possible links among local food webs). The strength of species migration between local food web patches is given as *M* (see Methods). In the most analyses it was assumed that habitats are heterogeneous and there are no within-species parameter correlations among habitats; yet, analyses with within-species correlations were also conducted to examine the role of habitat heterogeneity (see Methods). Random networks are assumed for both food web and habitat structure, and community stability is evaluated by local stability^3^ (the tendency for a community composition to return to the original equilibrium after a small perturbation). These settings allow direct comparison of the present study with previous studies.

## Results

**Figure 1:**
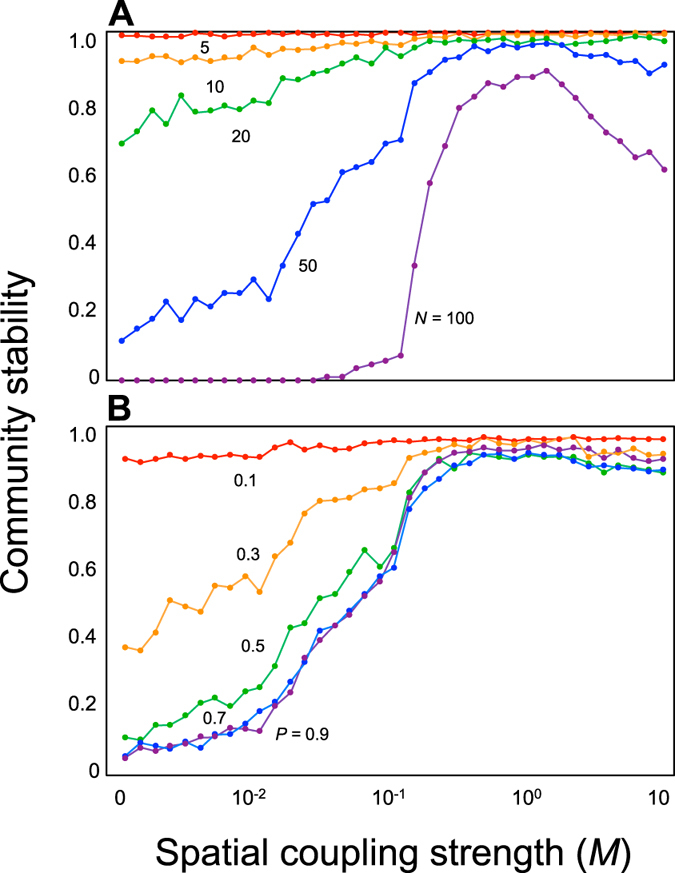
Relationships between the spatial coupling strength (*M*) and stability. (**A**) Effect of species richness (*N*). We assume *P* = 0.5. (**B**) Effect of proportion of connected pairs (*P*). We assume *N* = 50. Colours indicate different species richness and proportion of connected pairs. *s*_*il*_ is set to a random value from [0, 1]. *H*_*N*_ = 2, and *H*_*P*_ = 1.

We begin with the simplest case of two local food webs (*H*_*N*_ = 2, *H*_*P*_ = 1). When the local food webs were isolated (*M* = 0), increasing the food-web complexity tended to destabilize the population dynamics (Fig. 1), consistent with earlier theoretical studies^3^. When the two local food webs were coupled by migration (*M* > 0), the stability of the complex food web increased to that of a simple food web, revealing the stabilizing force of spatial complexity. Stabilization due to migration occurs as long as within-species correlations among habitats are not too strong (Supplementary Fig. S1). Stabilization effects of migration can thus be explained by the facts that 1) different communities may have different equilibria, and 2) immigration from a denser to a less dense population can act as a mechanism of stabilizing self-regulation (the diagonal elements of Jacobian community matrix becoming more negative as *M* increases; see Methods).

The magnitude of the stabilizing effect was dependent on the food-web complexity. Whereas for species-poor food webs the stability is already high without migration and thus the increase in stability arising from increasing coupling was limited, the stability of larger food webs showed a sharp, unimodal response to increased coupling strength (Fig. 1). This response suggests that stabilization arising from local food-web coupling is more prominent in a larger, more complex ecosystem, and that stabilization will not arise when the coupling is so strong that the whole meta-food web behaves as a single food web. These results were qualitatively unchanged by changing the type of network or functional response (Supplementary Figs S2, S3).

Next, we consider a case with more than three local food webs, to investigate the role of meta-community complexity. Our analysis revealed that meta-community complexity (*H*_*N*_, *H*_*P*_) affects the system stability in two interesting ways.

**Figure 2:**
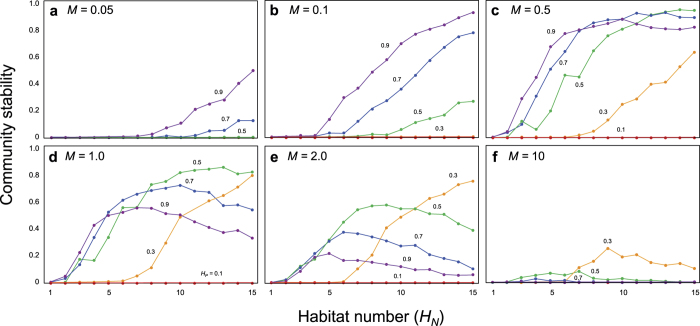
Relationship between the spatial complexity (*H*_*N*_ and *H*_*P*_) and stability with varying spatial coupling strength (*M*). *s*_*il*_ is set to a random value from [0, 0.1]. *N = *20. *P = *0.5.

First, meta-community complexity itself can stabilize community dynamics under intermediate coupling strength (*M*) (Fig. 2). Consider a complex food web that is unstable in isolation. When local food webs were loosely coupled (intermediate *M*), the community stability increased with an increasing number of local food webs (*H*_*N*_) or increasing connection probability (*H*_*P*_) (Fig. 2a–c). However, when the coupling between local food webs was too tight (larger *M*), the community stability showed a unimodal response to a changing number of local food webs (*H*_*N*_) or connection probability (*H*_*P*_) (Fig. 2d–f). Two mechanisms, (1) isolation of local food webs and (2) lowered average number of connected webs per local web are necessary to give rise to the pattern presented in Fig. 2. Which mechanism played a more critical role would be dependent of the number of local food webs, *H*_*N*_, as whether isolated local food webs exist or not depends on *H*_*N*_ and *H*_*P*_. An isolated complex food web has virtually no chance to be stable. The instability for lower *H*_*P*_ would be attributable solely to mechanism 1, as the probability that at least one isolated local food web exists is higher for lower *H*_*P*_. When *H*_*P*_ is larger, while there is non-negligible probability that isolated local food webs are present, changing *M* alters the relationship between *H*_*N*_ and community stability, implying that both mechanisms 1 and 2 jointly affects the community stability. Yet, for even larger *H*_*N*_ and *H*_*P*_, the probability that isolated local food web exists becomes negligible and the community stability would be determined mainly by the average number of connected webs.

**Figure 3:**
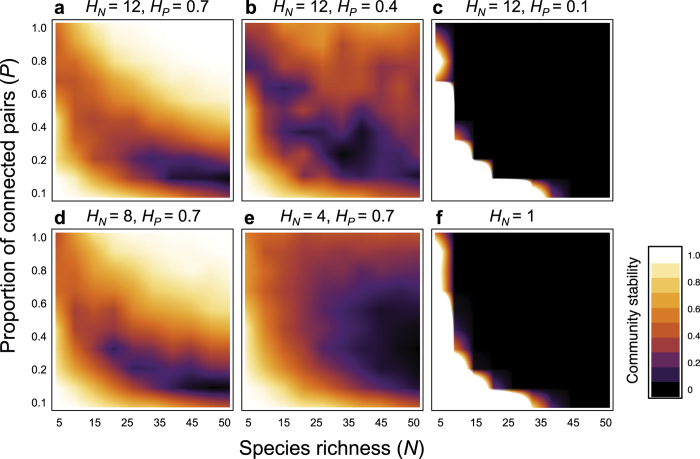
Complexity-stability relationships with varying spatial complexity (*H*_*N*_ and *H*_*P*_). *s*_*il*_ is set to a random value from [0, 0.1]. We assume *P* = 0.5 and *M = *1.

Second, meta-community complexity can reverse an otherwise negative complexity-stability relationship of food webs into a positive relationship (Fig. 3c,e,f). The reversal was observed as long as food webs were coupled by intermediate migration (Supplementary Figs S4–7). The positive complexity-stability relationship was more prominent in a community with more local food webs and connected pairs (Fig. 3a,b,d), suggesting that meta-population complexity is necessary for the stabilizing effect of food-web complexity.

## Discussion

Spatial heterogeneity in species composition among local food webs is necessary for the stabilizing effect to occur (Supplementary Fig. S1), because passive movement from high- to low-density local food webs generates the self-regulating, stabilizing effect. If the population densities of local food webs were too similar, then migration would not occur between the local food webs. Thus, the increased meta-community complexity would have virtually no effect on community stability (Supplementary Fig. S1). This observation is consistent with the widely accepted hypothesis that spatial heterogeneity can enhance the coexistence of many species^29^,^30^. However, the present mechanism differs from earlier hypotheses stating, for instance, that spatiality allows multi-species coexistence via spatial segregation or niche differentiation, or that different stages of ‘succession’ are maintained in the meta-community.

Our results demonstrate the effects that habitat destruction and modifications have on the ecological community. Consider species that are maintained in a meta-food web comprised of various local habitats. Habitat destruction can have three potential effects on the meta-community structure. First, habitat destruction can decrease the number of local food webs (lower *H*_*N*_). Second, it can disconnect pairs of local food webs by making it difficult for animals to move between habitats (lower *H*_*P*_). Third, even if the meta-community complexity is not altered, habitat modification can lead to loss of heterogeneity. Any of these changes has the potential to destabilize the ecosystem. Furthermore, once species loss is triggered, it can lead to cascading extinctions because the population dynamics are stabilized by the food-web complexity itself, which is supported by high species diversity.

Recently, a meta-community framework has been developed to understand what maintains the “unstable” complex food web^26^,^27^,^28^. With this framework, many local and simple sub-webs (e.g. linear food chain) are maintained via patch dynamics and a complex food web only emerges at a larger spatial scale as a spatial aggregation of local sub-webs. Our theory, predicting that complex food webs can be stably maintained even in local habitats, combined with the spatial-aggregation view, suggests a possibility that an extremely complex food web may be realized at a meta-food web level. It is important, however, that one would not be able to explain what maintains the complex food web, if the meta-community process is overlooked and the system is modelled as a single, complex food web.

The complexity of ecological communities, such as species diversity, has been of primary interest in understanding the role of biodiversity in ecosystem maintenance, and has been a major focus of biodiversity conservation^31^. Although the spatial role of ecosystem stability has mainly been understood in simple communities, it might be more important in complex communities. If complex communities are mutually supported by inherently unstable local communities, then greater attention may need to be given to both the species-connecting interaction network structure and the local community-connecting spatial network structure.

## Methods

Consider a random food web in which each pair of species, *i* and *j* (*i*, *j = *1,…, *N*), is connected by a trophic interaction with probability *P*. The maximum link number, *L*_max_, is *N*(*N* – 1)/2. The spatial food web model is defined by using the following ordinary differential equation:


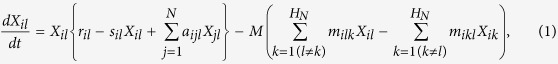


where *X*_*il*_ (*l = *1…*H*_*N*_) is the abundance of species *i* in habitat *l*, *r*_*il*_ is the intrinsic rate of change of species *i* in habitat *l*, *s*_*il*_ is density-dependent self-regulation of species *i* in habitat *l*, and *a*_*ijl*_ is the interaction coefficient between species *i* and species *j* in habitat *l*. Interaction coefficients are defined as *a*_*ijl*_* = e*_*ijl*_*α*_*ijl*_ and *a*_*jil*_ = −*α*_*ijl*_, where *α*_*ijl*_ is the consumption rate and *e*_*ijl*_ (<1) is the conversion efficiency. The migration rate is the product of a scaling parameter, *M* (spatial coupling strength), and the species-habitat specific migration rate, *m*_*ilk*_, where *k = *1…*H*_*N*_ but *k≠l*. For simplicity, we assume that *m*_*ilk*_ = *m*_*ikl*_. In the most analyses equilibrium species abundance *X*_il_^*^ and parameters *s*_*il*_, *e*_*ijl*_, *α*_*ijl*_, and *m*_*ilk*_ are randomly chosen from a uniform distribution, U[0, 1]. *r*_*il*_ is calculated such that *dX*_*il*_/*dt* = 0 for all *i* and *l*^32^. This setting means absence of within-species parameter correlations among habitats (i.e., heterogeneous habitats). Yet, this assumption can be relaxed. In two-habitat model, for example, we can control the correlations between the same parameter in two habitats, *x*_*1*_ and *x*_*2*_ (*X*_*il*_ and other parameters except for *m*_*ilk*_) by using *x*_*2*_ = *ρx*_*1*_ + 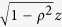, where *x*_*1*_ and *z* are the absolute values of random variables from standard normal distribution, and *ρ* is the correlation coefficients (0 < *ρ < *1).

Using stability analysis based on a Jacobian community matrix following May’s approach^3^, we calculate the community stability as the probability of the local equilibrium stability, which is estimated as the frequency of locally stable systems across 1000 sample communities^32^. We consider the consequences of a small perturbation in the equilibrium of the population dynamics model governed by equation 1. Dynamics of small deviations, *x*_*il*_, away from the equilibrium point, *X*_il_^*^, is given by:


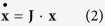


where 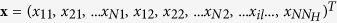 (*i = *1…N; *l* = 1…*H*_*N*_) and 
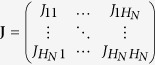
. Submatrices of the Jacobian matrix (J), *J*_*ij*_, are *N* × *N* matrices. Diagonal submatrices, *J*_*jj*_, are Jacobian matrices of *j*-th local food web in isolation. The diagonal and off-diagonal elements are represented as 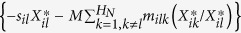 and *e*_*ijl*_*α*_*ijl*_*X*_il_^*^ (when sp. i consumes sp. j) or −*α*_*ijl*_*X*_il_^*^ (when sp. j consumes sp. i), respectively. The off-diagonal submatrices *J*_*ij*_ (*i≠j*) are the Jacobian matrices representing the effect of movements between local food webs. The diagonal and off-diagonal elements are *Mm*_*ilk*_, and zero, respectively.

## Additional Information

**How to cite this article**: Mougi, A. and Kondoh, M. Food-web complexity, meta-community complexity and community stability. *Sci. Rep.* **6**, 24478; doi: 10.1038/srep24478 (2016).

## Supplementary Material

Supplementary Information
