# Supplementary material for: Food-web complexity, meta-community complexity and community stability
Source: Sci Rep. 2016 Apr 13;6:24478. doi: 10.1038/srep24478 (PMC4829910; doi:10.1038/srep24478)

Supplementary Information

Food-web complexity, meta-community complexity and community stability

A. Mougi and M. Kondoh

Figure S1: Relationships between spatial coupling strength (M) and stability, with varying species richness (N), in six models with different levels of intra-species correlation in species abundance (X_{il}), intrinsic rate of change (r_{il}), interaction coefficients (a_{ij}) and density-dependent self-regulation (s_{il}) between local food webs (indicated with different colours). $P = 0.5$, $H_N = 2$ and $H_P = 1$.

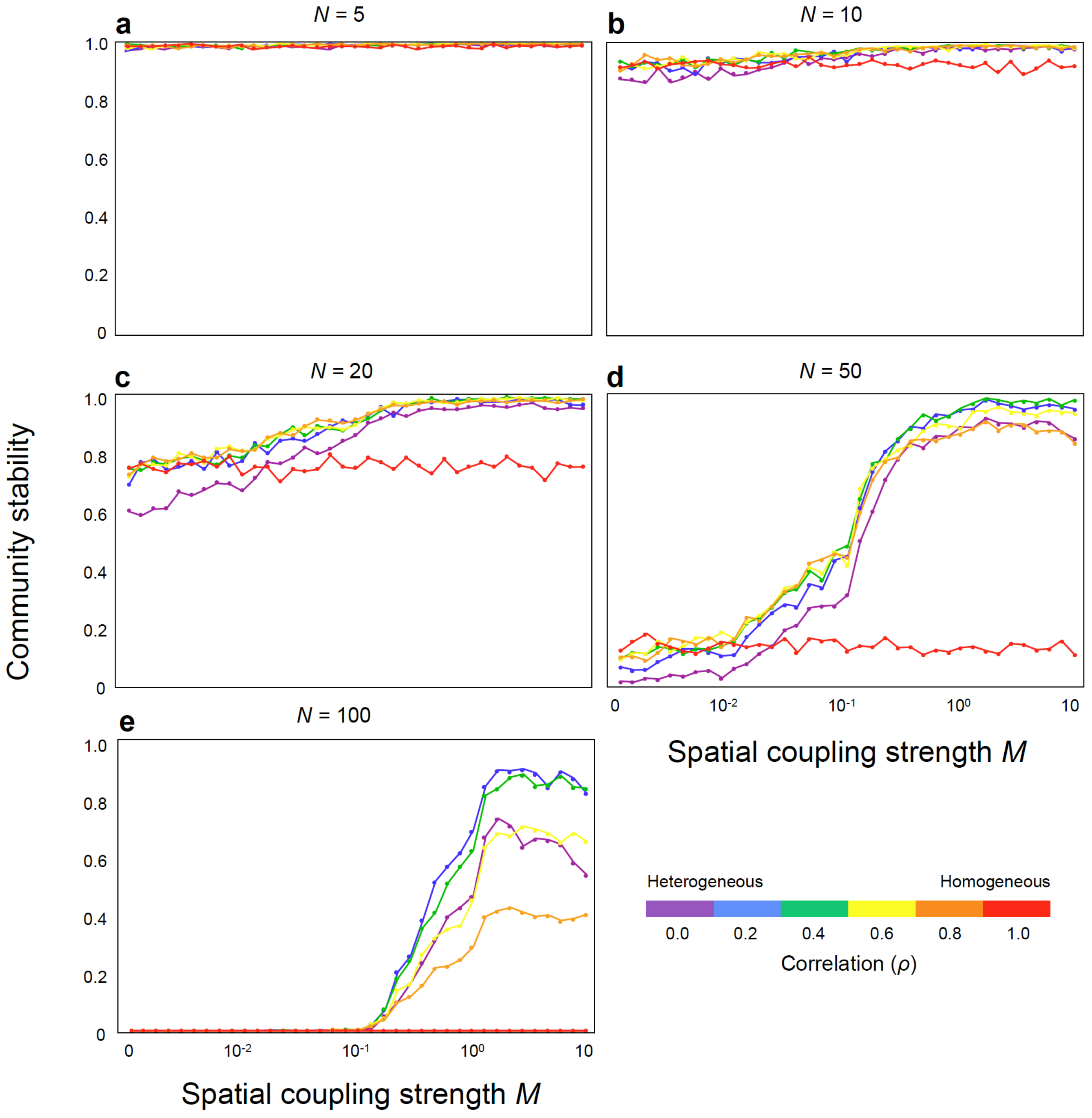

Figure S2: Relationships between spatial coupling strength (M) and stability, with varying species richness (N), in four models with different network structures and predator-type functional responses. In the cascade model, for each pair of species, $i, j = 1,\ldots, n$ with $i < j$, species i never consumes species j, whereas species j may consume species i. A type II functional response is given by

$$a_{ijl} = \sum_j e_{ijl}\,\alpha_{ijl}\,X_{jl} \Bigg/ \left(1 + \sum_k h_{ikl}\,\alpha_{ikl}\,X_{kl}\right),$$

where α_{ijl} is the resource consumption rate, e_{ijl} is the conversion efficiency, and h_{ijl} is the handling time. Different colours indicate different numbers of species. In the model with the type II functional response, s_{il} is randomly sampled from a uniform distribution between 0 and 0.25, because larger values of s_{il} stabilize the system even in the absence of migration. $P = 0.5$, $H_N = 2$ and $H_P = 1$.

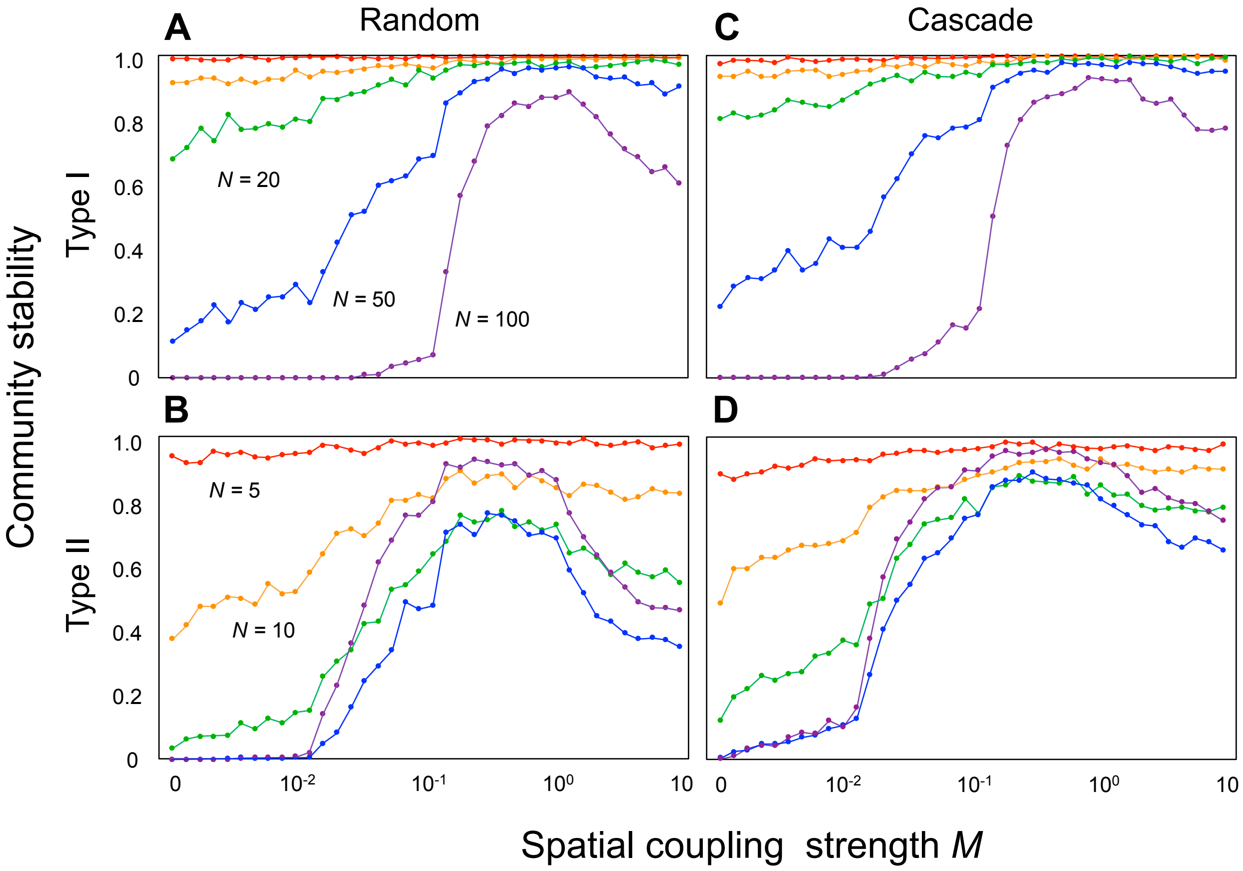

Figure S3: Complexity-stability relationship with varying spatial coupling strength (*M*) in two models with different network structures. We assume a type II functional response. s_{il} is randomly sampled from [0, 0.25]. In A and B, we assume $P = 0.5$. In C and D, we assume $N = 200$. We assume $H_N = 2$ and $H_P = 1$. Colours indicate different strengths of spatial coupling.

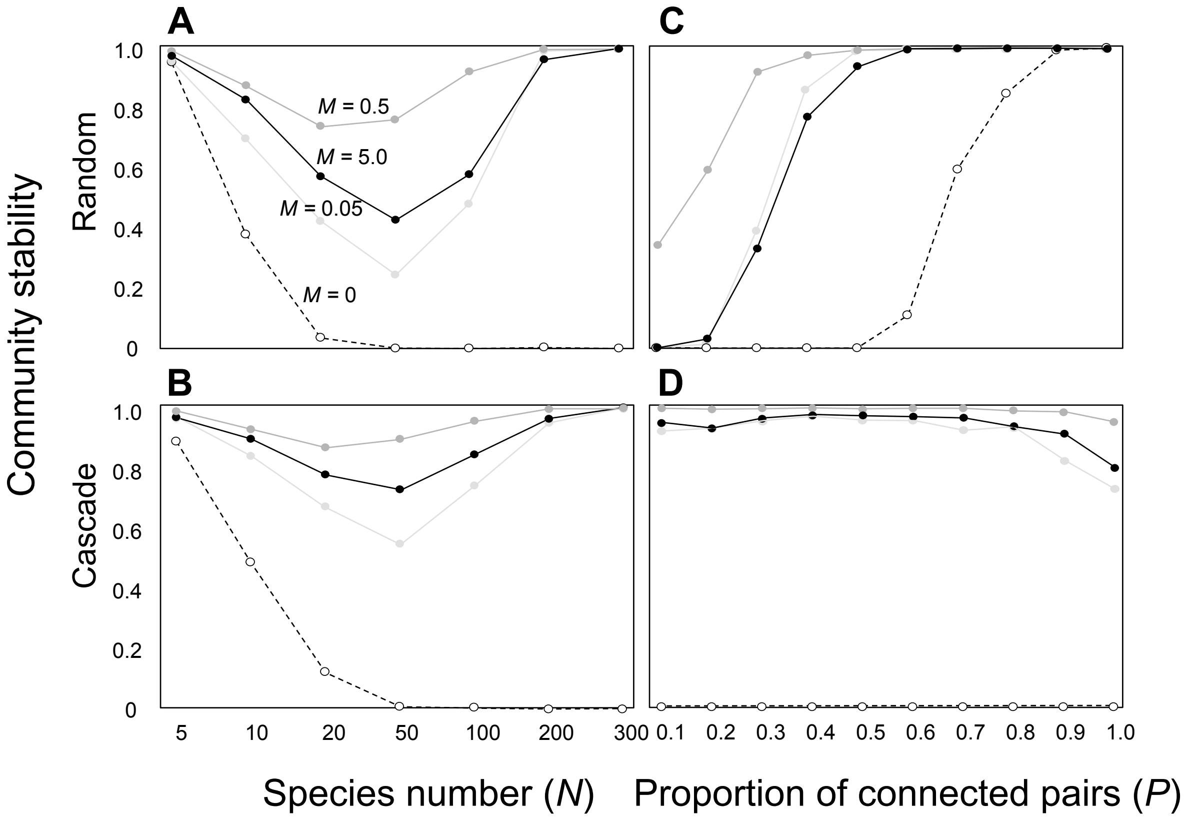

Figure S4: Relationship between habitat number (H_N) and stability, with varying species richness (N). Panels have different values of M. s_{il} is set to a random value in [0, 0.1]. $P = 0.5$ and $H_P = 0.7$.

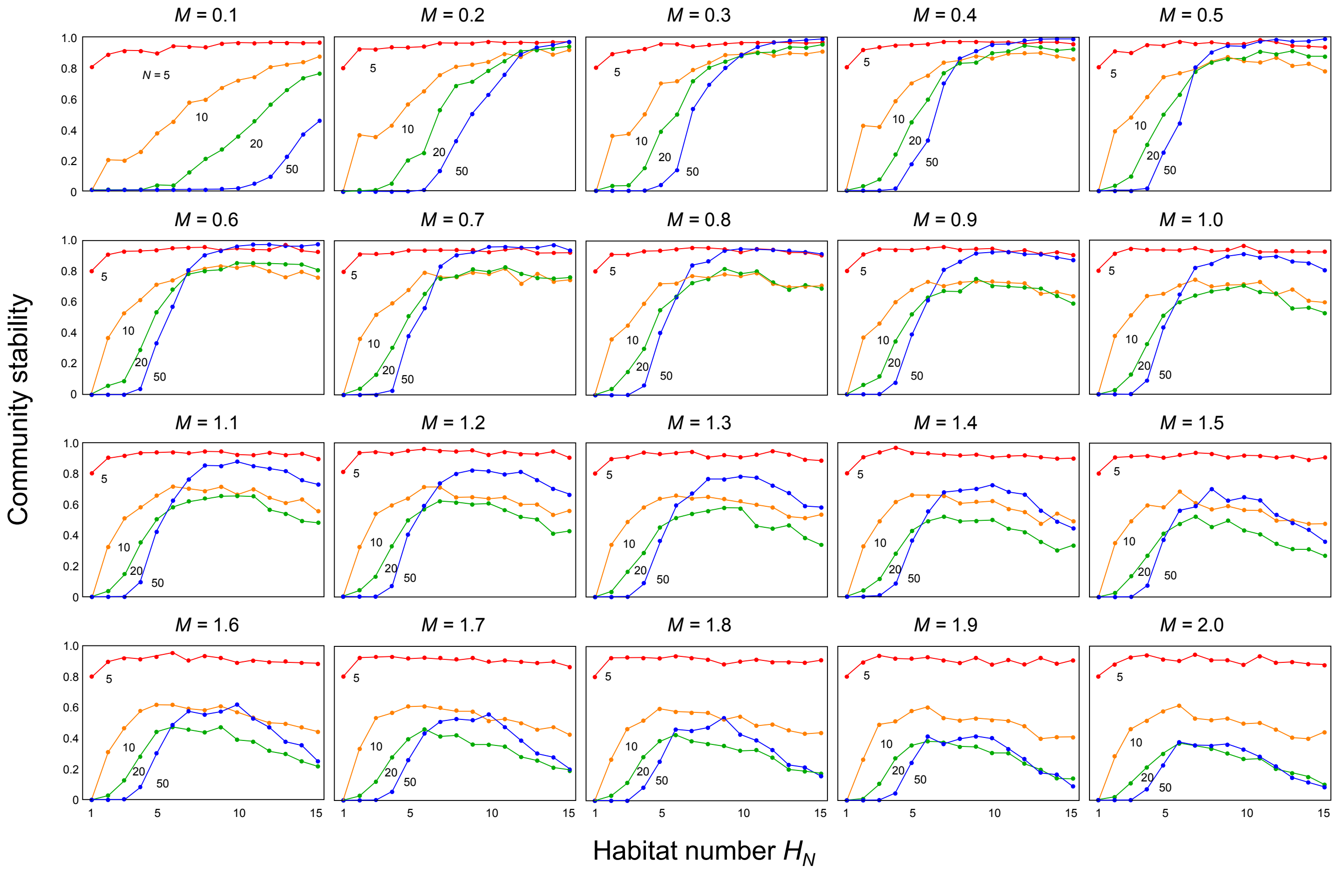

Figure S5: Relationship between habitat number (H_N) and stability, with varying proportions of connected pairs (P). Panels have different values of M. s_{il} is set to a random value in [0, 0.1]. $N = 20$ and $H_P = 0.7$.

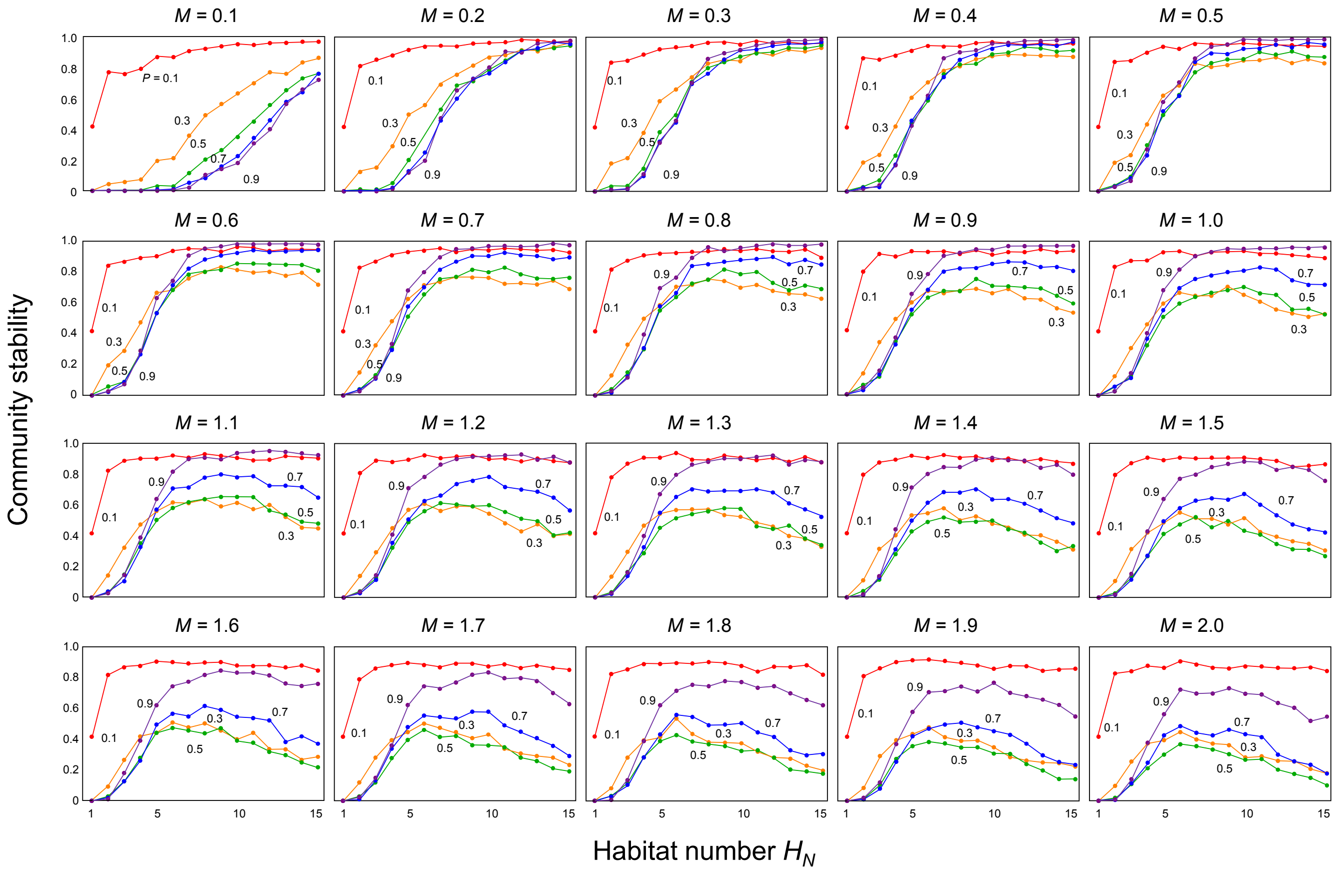

Figure S6: Relationship between the proportion of connected habitat pairs (H_P) and stability, with varying species richness (N). Panels have different values of M. s_{il} is set to a random value in [0, 0.1]. P = 0.5 and H_N = 10.

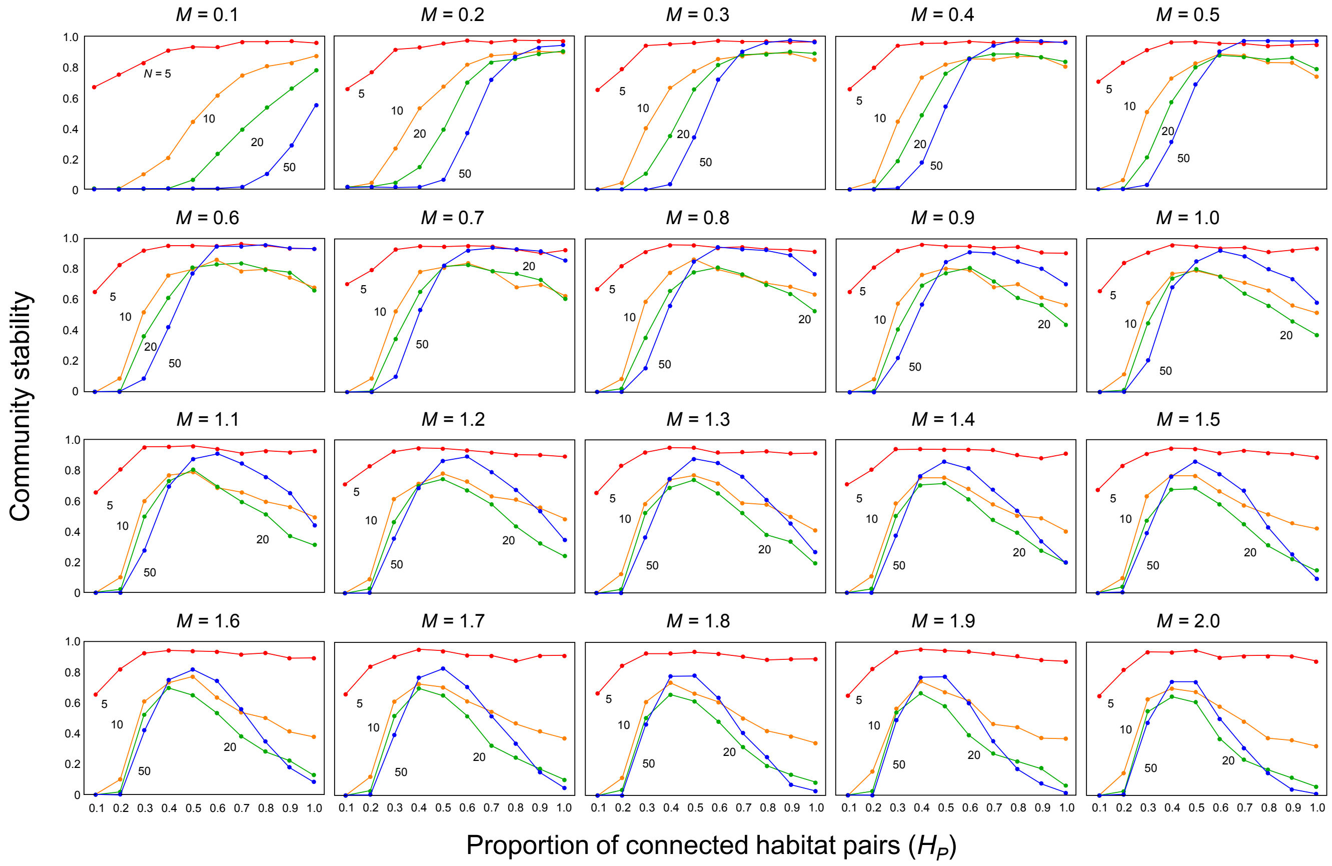

Figure S7: Relationship between the proportion of connected habitat pairs (H_P) and stability, with varying proportions of connected pairs (P). Panels have different values of M. s_{il} is set to a random value in [0, 0.1]. $N = 20$ and $H_N = 10$.

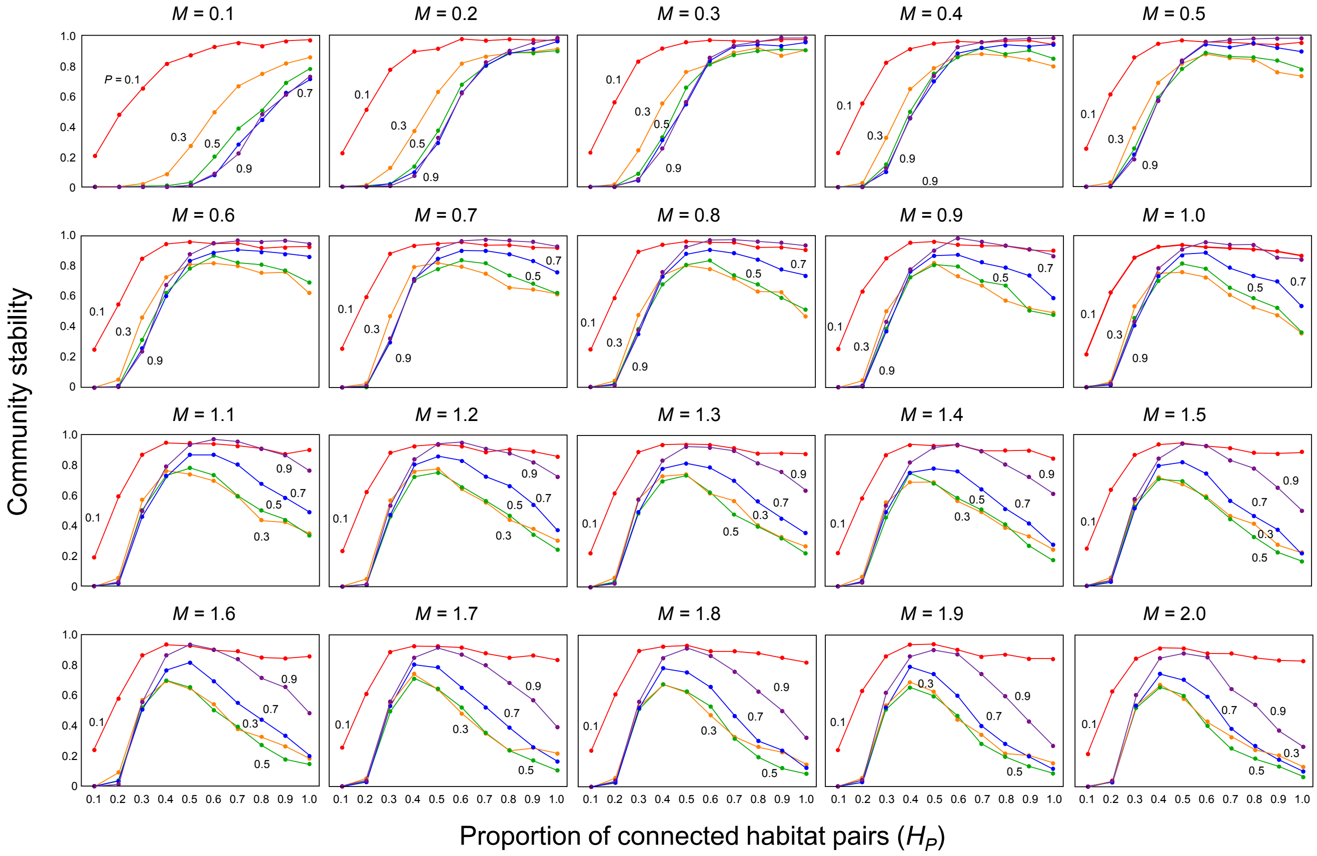

Supplement: Supplementary Information [file srep24478-s1.pdf]
